# Effects of Traditional Chinese Exercises on Cognitive Function in Older Adults With Mild Cognitive Impairment: A Systematic Review and Meta-Analysis

**DOI:** 10.3389/fnhum.2022.849530

**Published:** 2022-03-25

**Authors:** Kaixiang Zhou, Meng Liu, Dapeng Bao, Junhong Zhou

**Affiliations:** ^1^Sports Coaching College, Beijing Sport University, Beijing, China; ^2^College of Sports, Chengdu University of Traditional Chinese Medicine, Chengdu, China; ^3^China Institute of Sport and Health Science, Beijing Sport University, Beijing, China; ^4^Harvard Medical School, Hebrew SeniorLife Hinda and Arthur Marcus Institute for Aging Research, Boston, MA, United States

**Keywords:** traditional Chinese exercises, older adults, cognitive function, Tai Chi, Qigong, mild cognitive impairment

## Abstract

**Background:**

Recently, considerable research has been conducted to study the effects of traditional Chinese exercises (TCEs) on cognitive function in older adults with MCI. We completed a comprehensive systematic review and meta-analysis to assess the efficacy of TCEs on cognitive function in this population.

**Methods:**

A search strategy based on the PICOS principle was used to find the literatures in the databases of PubMed, Web of Science, MEDLINE, SPORT-Discus, PsycINFO, Cochrane Central Register of Controlled Trials, Ovid. The quality and risk of bias in the studies were independently assessed by two researchers.

**Results:**

Nine trials with 1,290 participants were included. The effect size of TCEs on global cognitive function was small (SMD = 0.29, 95% CI 0.15–0.44, *p* < 0.001) when compared to the active control and was moderate (SMD = 0.58, 95% CI 0.21–0.94, *p* = 0.002) compared to the inactive control; statistically significant effects were also found for short-term memory (SMD = 0.22, 95% CI 0.05–0.39, *p* = 0.013), long-term memory (SMD = 0.53, 95% CI 0.20–0.86, *p* = 0.002), shifting (SMD = −0.39, 95% CI −0.54 to −0.25, *p* < 0.001), language ability (SMD = 0.32, 95% CI 0.13–0.51, *p* = 0.001), visuospatial perception (SMD = 0.31, 95% CI 0.15–0.46, *p* < 0.001).

**Conclusion:**

This meta-analysis provides clinicians with moderate evidence to recommend that TCEs hold potential to enhance both global cognitive function and multiple domains of cognitive function, which, however, needs to be confirmed and further examined in futures studies. The results of this work provide critical knowledge for the design of future studies implementing TCEs as well as its clinical practice. Future RCTs with rigorous designs are needed to help obtain more definitive conclusions on the effects of TCEs on cognitive function in older adults with MCI.

## Introduction

Mild cognitive impairment (MCI) is a highly prevalent condition in which people demonstrate cognitive impairment with minimal impairment of instrumental activities of daily living (Petersen et al., 1999, 2014, 2018; Petersen, 2004). MCI significantly diminishes the life quality and functional independence in older adults, and has been linked to increased morbidity of dementia and mortality (Li et al., 2016; Hussenoeder et al., 2020). Multiple types of therapies and interventions have been developed for restoring cognitive function in older adults with MCI (Petersen et al., 2018). In addition to pharmacological interventions, more and more research efforts have been put on developing the non-pharmacological rehabilitative strategies for MCI (Petersen et al., 2018). A recent update practice guideline on MCI includes a recommendation for regular physical exercise as important part of non-pharmacologic treatments (Petersen et al., 2018). Two meta-analyses revealed that multi-component exercise tended to be the most effective exercise therapy for preventing the decline of global cognition in patients with MCI (Huang et al., 2021; Meng et al., 2021), but one showed the effects of physical exercise on cognitive function of older adults with MCI were small (SMD = 0.18∼0.35) (Biazus-Sehn et al., 2020). Additionally, studies also showed that many of physical exercises (i.e., moderate to high intensity aerobic and strength exercises) induced limited benefits for cognitive function with frequently reported adverse events in older adults with MCI due to the high intensity of intervention and/or insufficient safety procedure (Lamb et al., 2018; Devenney et al., 2019). Therefore, the exercise with mild-to-moderate physical intensity and sufficient safety is highly demanded.

The traditional Chinese exercises (TCEs) are a kind of multimodal mind-body exercise derived from traditional Chinese medicine, by targeting multiple components simultaneously, including aerobic, postural balance control, cognitive, social, and meditation elements (Jiang and Zou, 2013; Chang et al., 2014; Wei et al., 2020). Studies have shown that TCEs can help promote the blood circulation, regulate the internal organs, activate the muscles and tendons, and regulate the breath pace (Ho et al., 2011; Janelsins et al., 2011; Wayne et al., 2012; Jiang and Zou, 2013; Chang et al., 2014). TCEs are with mild-to-moderate physical intensity and the performance of it is not restricted by the condition of equipment, manpower, and venue. The TCEs have been widely used for the rehabilitation of physical function or help prevent falls in older adults (Taylor-Piliae et al., 2014; Li et al., 2019; Liu et al., 2020). Recently, studies have emerged to explore the effects of TCEs on cognitive function in older adults with MCI (Cai et al., 2020; Ye et al., 2020; Li et al., 2021; Wang et al., 2021). However, the results on the effects of TCEs on cognitive function were inconsistent. For example, one study showed that 12 months of Tai Chi did not significantly improve global cognitive function in older adults with MCI (Lam et al., 2014); while another one showed that 3 months of Tai Chi could significantly improve global cognitive function in older adults with MCI (Birimoglu Okuyan and Deveci, 2020). Even the two previously published meta-analyses showed different findings. Specifically, Zhang et al. (2019) showed that TCEs (Tai Chi or Liuzijue) cannot benefit either global cognitive functions or other specific cognitive domains (e.g., memory, executive function, verbal fluency), but Wei et al. (2020) concluded that the Tai Chi induced large benefits on global cognitive ability and long-term delayed recall ability. These inconsistencies in the original studies or meta-analysis may arise from the variance in study design and the methodology of analysis. For example, the meta-analysis completed by Wei et al. (2020) focused on Tai Chi but only using mean difference (MD), which may not be an appropriate measure of the effect size, while Zhang et al. (2019) included different types of the TCEs but with relatively small sample size (*n* = 5). Additionally, the inclusion of non-randomized and -controlled trials may contribute to the high heterogeneity of study results (Wei et al., 2020); and the characteristics of TCEs intervention (e.g., duration of intervention, type of control group) were overlooked and the influence of these factors on the benefits of TCEs have not been well examined.

Therefore, to highlight the most recent study findings in this field and to explicitly examine the effects of TCEs on global cognitive function and the specific cognitive domains in older adults with MCI, we here conducted a systematic review and meta-analysis based upon the peer-reviewed publications. Only randomized controlled trials (RCTs) were included in this work, and several subgroup analyses, such as the protocol of interventions (e.g., intervention duration) were performed to provide critical knowledge of the appropriate intervention design, informing future’s studies with rigorous design.

## Methods

This systematic review and meta-analysis was conducted using Preferred Reporting Items for Systematic Reviews and Meta-Analysis guidelines (Higgins et al., 2019) and registered with PROSPERO (Registration ID CRD42021289913), an international prospective registry for systematic reviews.

### Data Sources and Search Strategies

Two authors (KZ and ML) independently searched PubMed, Web of Science, MEDLINE, SPORT-Discus, PsycINFO, Cochrane Central Register of Controlled Trials, and Ovid databases from inception to January 28, 2022 using a comprehensive search strategy (Supplementary Material 1). A secondary search strategy was also used which involved a manual search in the reference lists of selected articles. Searches were limited to English language only and no date restrictions were applied.

### Selection Criteria

Studies were included if they met the following criteria: (1) the participants were of mean age ≥ 60 years and were assessed as MCI by clinicians with validated tools in appropriate scenarios (Petersen et al., 2018); (2) the intervention used was single TCEs, including Tai Chi, Baduanjin Qigong, Wuqinxi Qigong, Liuzijue Qigong; (3) the control included usual care, health education or no intervention but also active comparison conditions (e.g., stretching, aerobic exercises); (4) the outcomes were global cognitive function (primary outcome) or at least one specific cognitive domain (secondary outcomes) assessed by neuropsychological tests or other objective measurement; the classification of specific cognitive domain was followed by previous studies (Chen et al., 2020; Diamond, 2020; Ren et al., 2021); (5) the design of study was randomized controlled trial.

Articles were excluded if: (1) the language was non-English or unable to obtain outcome data; (2) review papers and conference articles; (3) repeated publications.

### Data Extraction

The process of data extraction was conducted independently by two authors (KZ and ML) according to the Cochrane Collaboration Handbook (Higgins et al., 2019). The extracted information of the publications included: study (authors, year), participants (age, sex, educational level), grouping and sample size, interventions (type, frequency, number of sessions, time length of each session, intervention duration), environment (e.g., group classes and/or home practices), and outcome measures. When one study consisted of multiple outcome measures characterizing one cognitive domain (e.g., MMSE, MoCA, and ADAS-Cog all measure global cognitive function), only one outcome measure was used and included in the analysis. Any outcome measures on which the two authors disagreed was discussed with other two authors (JZ and DB) until a consensus was achieved.

For each included study, the mean and standard deviation of each outcome in post-tests were extracted. If these values were not available, they were calculated using the following formulas, where the correlation coefficient (Corr) was set at 0.5 (Higgins et al., 2019; Xue et al., 2019).


Meanpost=Meanpre+Meanchange

$$\text{SDpost}=\frac{\begin{array}{c} 2\times \mathrm{Corr}\times \text{SDpre}\;\\ +\sqrt{4\times \text{Corr}2\times \text{SDpre}2-4\times \left(\text{SDpre}2-\text{SDchange}2\right)}\; \end{array}}{2}$$

If any relevant data was missing, we tried to contact the corresponding author or other authors of that study via email to request it (Higgins et al., 2019).

### Quality Assessment

The quality of included studies was assessed independently by two authors (KZ and ML) based on the principles of the Physiotherapy Evidence Database (PEDro). The PEDro scale includes 11 items, and each study was assessed as either “yes” (score 1) or “no” (score 0) for each of those items. According to the PEDro guidelines, the maximum total score was 10 (item 1 is not used to compute the total score). If a study received a score of 9 or 10, it was considered to be of very good quality; a score of 6–8 reflected good quality; a score of 4 or 5 showed medium quality; and a score of 0–3 suggested poor quality (Maher et al., 2003). The quality of the evidence was also assessed independently by two authors (KZ and ML) based on the GRADE criteria (Andrews et al., 2013a,b). Any score on which the two authors disagreed was discussed with a third author (JZ and DB) until a consensus was achieved.

### Statistical Analysis

To determine the effect size (ES) of the intervention, the standardized mean differences (SMDs) of the outcomes were calculated. Effect sizes were classified as trivial (<0.2), small (0.2∼0.5), moderate (0.5∼0.8), or large (>0.8) (Cohen, 1988). Meta-analysis was performed in Stata v15.1 (STATA Corp., College Station, TX) using the inverse variance method for included studies that compared the effects of TCEs and control conditions on each included outcome. Statistical heterogeneity was evaluated using heterogeneity chi-squared (χ^2^) and *I*^2^-values. The level of heterogeneity was interpreted according to guidelines from the Cochrane Collaboration: *I*^2^-values of 25, 50, and 75% for low, moderate, and high heterogeneity, respectively (Higgins et al., 2003). A random-effect model was used to conservatively estimate the pooled effect in anticipation of heterogeneity across the studies due to differences in participants and intervention characteristics. In addition, publication bias was assessed by generating funnel plots and conducting Egger’s test. If a significant asymmetry was detected, we used Trim and Fill method for sensitivity analysis of the results (Duval and Tweedie, 2000).

To examine characteristics that may affect TCEs efficacy, we performed subgroup analyses using the random-effect model. Analyses were performed for overall outcomes based on the following study characteristics: control type (i.e., active or inactive), intervention type (Tai Chi, Baduanjin, Wuqinxi, Liuzijue), sessions length (i.e., short-to-moderate length < 24 weeks or longer sessions length ≥ 24 weeks), education level of participants, intervention environment (i.e., group classes and/or home practices), diagnostic criteria for MCI. All the statistical significance was set at *p* < 0.05.

## Results

### Study Selection

**FIGURE 1:**
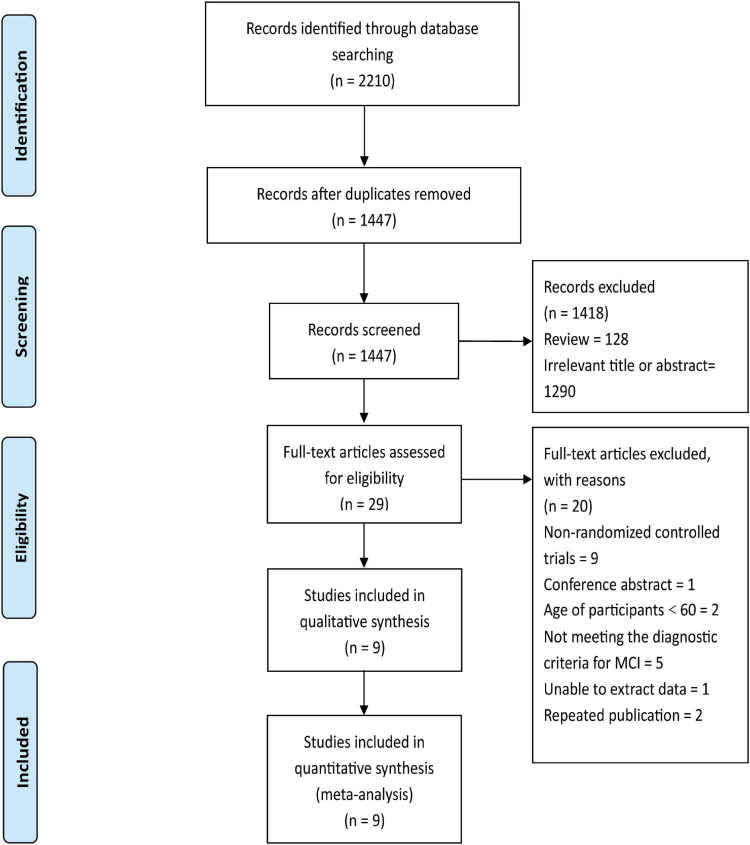
Flow chart for selection of studies.

The flow of the study identification and selection process is summarized in Figure 1. The initial search identified 2,210 potentially relevant articles (PubMed *n* = 151, PsycINFO *n* = 66, Web of Science *n* = 276, MEDLINE *n* = 160, Ovid *n* = 12, SPORT-Discus *n* = 17, The Cochrane Central Register of Controlled Trials *n* = 1,528). After the removal of duplicates, we identified 1,447 publication records, consisting of 29 publications with full-text. Next, these 29 full-text articles were evaluated for eligibility, and 19 of them were excluded. Therefore, after completing the thorough full-text review, 10 publications were included. In addition, one study was excluded because data could not be extracted and the original data provided by the authors were not available. Finally, nine publications (Table 1) consisting of 1,290 participants were included in the quantitative synthesis.

**TABLE 1:** Characteristics of the included studies (*n* = 9).

Study	Sample size	Mean age (Years)	%F	Educational level (Years)	Diagnostic criteria	Interventions	Group and/or home	Outcome measures
Lam et al. (2011)	Tai Chi (*n* = 171)	77.2	73.1	4.1	Mayo clinic diagnostic criteria/CDR (0.5)	24-form style Tai Chi: 30 min/session × 3 times/week × 16∼20 weeks (8–12 weeks induction phase and 8 weeks maintenance phase)	Both	Global cognition function: MMSE→ ADAS-Cog→; CDR ↑; R Short-term memory: Digit span→; Long-term memory: Delay recall→; Shifting: Chinese Trail A/B→; Language ability: Category verbal fluency→; Visuospatial perception: Visual span→; Others: BBS; MIC; NPI; CDS;
	Active-control (*n* = 218)	78.3	79.9	2.6		Stretching and toning exercises:30 min/session × 3 times/week × 16∼20 weeks		Outcome measured at baseline, 12th week, 20th week.
Lam et al. (2012)	Tai Chi (*n* = 171)	77.2	73.1	4.1	Mayo clinic diagnostic criteria/CDR (0.5)	24-form style Tai Chi: 30 min/session × 3 times/week × 52∼54 weeks (4–6 weeks induction phase and 1 year maintenance phase)	Both	Global cognition function: MMSE→; ADAS-Cog→; CDR ↑; Short-term memory: Digit span→; Long-term memory: Delay recall ↑; Shifting: Chinese Trail A/B→; Language ability: Category verbal fluency→; Visuospatial perception: Visual span→; Others: BBS; MIC; NPI; CDS.
	Active-control (*n* = 218)	78.3	79.9	2.6		Stretching and toning exercises:30 min/session × 3 times/week × 52∼54 weeks		Outcome measured at baseline, 20th week, 36th week, 54th week.
Sungkarat et al. (2017)	Tai Chi (*n* = 33)	68.3	94	11.4	Petersen diagnostic criteria	10-form Tai Chi: 50 min/session × 3 times/week × 15 weeks	Both	Short-term memory: Digit Spanry→; Long-term memory: LMDRS ↑; Shifting: TMT Part (B-A) ↑; Visuospatial perception: Block design ↑; Others: Edge-contrast sensitivity; Knee proprioception; Knee extension strength; Hand reaction time; Postural sway; PPA fall risk index scores.
	Inactive-control (*n* = 33)	67.5	79	9.3		Health education		Outcome measured at baseline, 15th week.
Sungkarat et al. (2018)	Tai Chi (*n* = 33)	68.3	94	11.4	Petersen diagnostic criteria	10-form Tai Chi: 50 min/session × 3 times/week × 24 weeks	Both	Short-term memory: Digit Span→; Long-term memory: LMDRSo ↑; Shifting: TMT Part (B-A) ↑; Visuospatial perception: Block design→; Others: Plasma Variables (BDNF/TNF-α/IL-10);
	Inactive-control (*n* = 33)	67.5	79	9.3		Health education		Outcome measured at baseline, 24th week.
Niu et al. (2019)	Qigong (*n* = 36)	>60	60	7	Participants were diagnosed with mild VCI, MoCA < 21, cognitive impairment lasted for more than 3 months	Breath Qigong:1 h daily for 12 weeks	Group	Global cognition function: MoCA ↑; LOTCA ↑;
	Qigong+Cognitive Training (*n* = 50)		35	6.7		Breath Qigong + Cognitive Training:1 h daily for 12 weeks		Others: Barthel ADL score
	Active-control (*n* = 40)		50	6.6		Cognitive training:1 h daily for 12 weeks		Outcome measured at baseline,12th week.
Birimoglu Okuyan and Deveci (2020)	Tai Chi (*n* = 23)	74.2	42.5	<5	MMSE < 25, MoCA < 25, and CDR (0.5∼1)	Yang style Tai Chi:35∼40 min/session × 2 times/week × 12 weeks	Group	Global cognition function: FB scale (cognitive adaptations) ↑; Others: Tinetti balance/gait; TSK; PASE;
	Inactive-control (*n* = 24)					Routine daily activities		Outcome measured at baseline,12th week.
Liu et al. (2021)	Qigong (*n* = 23)	66.2	75	11.2	Petersen diagnostic criteria, MoCA < 26, Lawton-Brody ADL score < 18, Global Deterioration Scale score at 2 or 3	Baduanjin:60 min/session × 3 times/week × 24 weeks	Group	Global cognition function: MoCA ↑;
	Active-control (*n* = 23)	64.3	58.8	10.6		Brisk Walking (55∼75%HRR): 60 min/session × 3 times/week × 24 weeks		Others: fMRI
	Inactive-control (*n* = 23)	66	70	11.5		Health education		Outcome measured at baseline,24th week.
Zheng et al. (2021)	Qigong (*n* = 23)	65.8	74	≥6	Petersen diagnostic criteria	Baduanjin:60 min/session × 3 times/week × 24 weeks	Group	Global cognition function: MoCA ↑;
	Active-control (*n* = 23)	64.9	52	≥6		Brisk Walking (55∼75%HRR): 60 min/session × 3 times/week × 24 weeks		Short-term memory: Digit Span→; Long-term memory: Delay recall→; Others: fMRI;
	Inactive-control (*n* = 23)	65.9	74	≥6		Health education		Outcome measured at baseline, 24th week.
Li et al. (2022)	Tai Chi (*n* = 23)	74.4	69.6	12 (52.2%), 11 (47.8%)	Petersen diagnostic criteria	Cognitively Enhanced Tai Chi:60 min/session × 2 times/week × 16 weeks	Group	Global cognition function: MoCA^#^
	Tai Chi (*n* = 22)	74.5	36.4	10 (45.5%), 12 (54.5%)		8-form Tai Chi: 60 min/session × 2 times/week × 16 weeks		Short-term memory: Digit span^#^ Shifting: Trail-Making B^#^ Language ability: Verbal fluency^#^ Others: Intervention feasibility, acceptability, and safety; Dual-task cost
	Active-control (*n* = 24)	74.9	62.5	13 (54.2%), 11 (45.8%)		Stretching:60 min/session × 2 times/week × 16 weeks		Outcome measured at baseline,16th week.

*ADAS-Cog, Alzheimer’s Disease Assessment Scale-Cognitive subscale; ADL, Activities of Daily Living score; BBS, Berg Balance Scale; CDR, Clinical Dementia Rating; CDS, Cornell Depression Score; FES, Falls Efficacy Scale; FB, Falls Behavioral; FAB, Frontal Assessment Battery at bedside; fMRI, functional Magnetic Resonance Imaging; HRR, Heart Rate Reserve; LOTCA, Loewenstein Occupational Therapy Cognitive Assessment; LMDRS, Logical Memory delayed recall score; MMSE, Mini-Mental State Examination; MoCA, Montreal Cognitive Assessment Scale; MIC, Memory Inventory for the Chinese; NPI, Neuropsychiatric Inventory; PSQI, Pittsburgh Sleep Quality Index; PPA fall risk index scores, Physiological Profile Assessment fall risk index scores; PASE, Physical Activity Scale for the Elderly; RBANS, Repeatable Battery for the Assessment of Neuropsychological Status; TMT Part (B-A), Trail-Making Test Part B-A; TMIG, Functional Capacity Tokyo Metropolitan Institute of Gerontology Index of Competence; TSK, Tampa Scale of Kinesiophobia; VCI, Vascular Cognitive Impairment; →, no significant difference between groups (p > 0.05); ↑, significant difference between groups (p < 0.05); #, No statistical difference was reported.*

### Quality Assessment

It was demonstrated that all the nine studies were scored between 6 and 8 (Table 2), suggesting the quality of the included studies was good. The experimental design of two studies did not adapt allocation concealment, one study did not report blinding, and eight studies implemented a single blinded (assessors blinded) trial protocol. The quality of evidence for the primary outcome (global cognition function) was moderate; and the quality of evidence for the secondary outcomes (short-term memory, long-term memory, shifting, language, ability, visuospatial perception) was low to moderate (Supplementary Table 1).

**TABLE 2:** Quality assessment of included studies (*n* = 9).

Study	Eligibility criteria	Random allocation	Concealed allocation	Similarity baseline	Subject blinding	Therapist blinding	Assessor blinding	>85% retention	Intention-to-treat	Between-group comparisons	Point and variability measures	Total score
Lam et al. (2011)	1	1	0	1	0	0	1	0	1	1	1	6
Lam et al. (2012)	1	1	0	1	0	0	1	0	1	1	1	6
Sungkarat et al. (2017)	1	1	1	1	0	0	1	1	1	1	1	8
Sungkarat et al. (2018)	1	1	1	1	0	0	1	1	1	1	1	8
Niu et al. (2019)	1	1	1	1	0	0	1	0	1	1	1	7
Birimoglu Okuyan and Deveci (2020)	1	1	1	1	0	0	0	1	1	1	1	7
Liu et al. (2021)	1	1	1	1	0	0	1	1	1	1	1	8
Zheng et al. (2021)	1	1	1	1	0	0	1	1	1	1	1	8
Li et al. (2022)	1	1	1	1	0	0	1	1	1	1	1	8

### Characteristics of Included Studies

#### Participant Characteristics

Both men and women were included in each of the included studies. These studies were conducted in four countries including United States (*n* = 1), Turkey (*n* = 1), Thailand (*n* = 2), and China (*n* = 5). The mean ages of the participants ranged from 60 to 78.3 years (Table 1). The education of participants ranged from 2.6 to 13 years. None of the participants across all the studies had previous experience with TCEs and had no regular exercise habit. In six of the studies (Lam et al., 2011, 2012; Sungkarat et al., 2017, 2018; Zheng et al., 2021; Li et al., 2022), participants were recruited from community centers; in another two studies (Niu et al., 2019; Birimoglu Okuyan and Deveci, 2020), participants were in an institutionalized environment (hospital or nursing home); and in the other one study, the living environment of the participants was not provided (Liu et al., 2021). Participants in two studies were diagnosed with amnestic mild cognitive impairment (aMCI) by Clinical Dementia Rating (CDR), or Mayo clinic criteria (Lam et al., 2011, 2012); five studies selected Petersen’s criteria (Petersen, 2004) for diagnosing aMCI (Sungkarat et al., 2017, 2018; Liu et al., 2021; Zheng et al., 2021; Li et al., 2022); participants with mild vascular cognitive impairment in one study were diagnosed by MoCA to determine whether or not the they had MCI (Niu et al., 2019); and another one study selected MMSE, MoCA, and CDR scale for diagnosing MCI (Birimoglu Okuyan and Deveci, 2020).

#### Intervention Characteristics

The information of the intervention parameters is included in Table 1. Six studies focused on Tai Chi and three studies focused on Qigong. The sub-types of Tai Chi included 24-form style Tai Chi (*n* = 2), 10-form Tai Chi (*n* = 2), 8-form Tai Chi (*n* = 1), and Yang style Tai Chi (*n* = 1). The sub-types of Qigong included breath Qigong (*n* = 1), and Baduanjin Qigong (*n* = 2). Four of the studies used active control. Specifically, two compared the effects between 24-form style Tai Chi and stretching exercises (Lam et al., 2011, 2012); one compared the effect between 8-form Tai Chi and stretching (Li et al., 2022); and another one compared the differences between breath Qigong and cognitive training and further explored the effects of breath Qigong combined with cognitive training (Niu et al., 2019). Three studies used the inactive control. Specifically, two studies compared the effects between 10-form Tai Chi and health education (Sungkarat et al., 2017, 2018); and the other one used the routine daily activities compared to Yang style Tai Chi (Birimoglu Okuyan and Deveci, 2020). Two studies used active control and inactive control to compare the effects among Baduanjin Qigong, brisk walking and health education (Liu et al., 2021; Zheng et al., 2021).

The duration of the intervention session varied from 30 to 60 min for nine studies. The frequency of the intervention was more than two times a week. Specifically, six studies conducted three sessions a week (Lam et al., 2011, 2012; Sungkarat et al., 2017, 2018; Liu et al., 2021; Zheng et al., 2021); two conducted two sessions a week (Birimoglu Okuyan and Deveci, 2020; Li et al., 2022); and another one conducted breath Qigong every day (Niu et al., 2019). The overall duration of the interventions ranged from 12 to 54 weeks and included 24∼162 sessions. Specifically, one study conducted 24 sessions of Yang style Tai Chi for 12 weeks (Birimoglu Okuyan and Deveci, 2020); one conducted 45 sessions of 10-form Tai Chi for 15 weeks (Sungkarat et al., 2017); one conducted 32 sessions of 8-form Tai Chi for 16 weeks (Li et al., 2022); one conducted 48∼60 sessions of 24-form Tai Chi for 16∼20 weeks (Lam et al., 2011); one conducted 72 sessions of 10-form Tai Chi for 24 weeks (Sungkarat et al., 2018); one conducted 152∼162 sessions of 24-form Tai Chi for 52∼54 weeks (Lam et al., 2012); one conducted 84 sessions of breath Qigong for 12 weeks (Niu et al., 2019); and the other two conducted 72 sessions of Baduanjin for 24 weeks (Liu et al., 2021; Zheng et al., 2021). Four studies (Lam et al., 2011, 2012; Sungkarat et al., 2017, 2018) used group classes plus home practice sessions for their interventions and five studies (Niu et al., 2019; Birimoglu Okuyan and Deveci, 2020; Liu et al., 2021; Zheng et al., 2021; Li et al., 2022) used group classes only.

#### Study Outcomes

Details of the outcomes in each study are summarized in Table 1. The global cognitive function was assessed in seven of the studies. Specifically, two studies used MMSE, Alzheimer’s Disease Assessment Scale-Cognitive subscale (ADAS-Cog), and Clinical Dementia Rating scale (Lam et al., 2011, 2012); three used MoCA scale (Liu et al., 2021; Zheng et al., 2021; Li et al., 2022); one used MoCA and Loewenstein Occupational Therapy Cognitive Assessment (LOTCA) (Niu et al., 2019); and another one used Falls Behavioral subscale (Birimoglu Okuyan and Deveci, 2020). The working memory was assessed in six of the studies. Specifically, one study assessed short-term memory with a digit span test (Li et al., 2022); three studies assessed short- and long-term memory using digit span test and delay recall test (Lam et al., 2011, 2012; Zheng et al., 2021); and the other two assessed short- and long-term memory using digit span test and logical memory delayed recall score (Sungkarat et al., 2017, 2018); The shifting was assessed in five of the studies. Specifically, two studies used Chinese Trail A or B (Lam et al., 2011, 2012); one selected Trail-Making Test Part B (Li et al., 2022), and the other two selected Trail-Making Test Part B-A (Sungkarat et al., 2017, 2018). The language ability was assessed by verbal fluency test in three of the studies (Lam et al., 2011, 2012; Li et al., 2022). The visuospatial perception was assessed in four of the studies. Specifically, two studies used visual span test (Lam et al., 2011, 2012); and the other two used block design test (Sungkarat et al., 2017, 2018). Interestingly, other characteristics related to cognitive function were also assessed. Specifically, one study used functional MRI to explore the effects of TCE on brain structure and function (Liu et al., 2021). The physical activity, balance, falls, mood, acceptability, and safety was also assessed in several studies (Lam et al., 2011, 2012; Sungkarat et al., 2017, 2018; Li et al., 2022).

To characterize global cognitive function, the scores of MMSE, MoCA, or Falls Behavioral subscale (cognitive adaptations) scores were included; To characterize the memory function, the performance of digit span test (short-term memory) and delay recall test (long-term memory) were incorporated; The shifting ability is characterized by trail making test; the language ability was measured by verbal fluency task; and the visuospatial perception was assessed by using visual span backward or block design scores.

#### Effects of Traditional Chinese Exercises on Global Cognitive Function

All the included studies showed that the TCEs can induce significantly greater improvement in global cognitive function as compared to the inactive control groups, but inconsistent results were observed when comparing the effects between TCEs and active control. Specifically, two of the included studies observed no significant difference between Tai Chi and stretching (Lam et al., 2011, 2012); and the other three studies observed that Qigong (Baduanjin or breath Qigong) induced significantly greater improvement as compared to brisk walking and/or cognitive training (Niu et al., 2019; Liu et al., 2021; Zheng et al., 2021).

**FIGURE 2:**
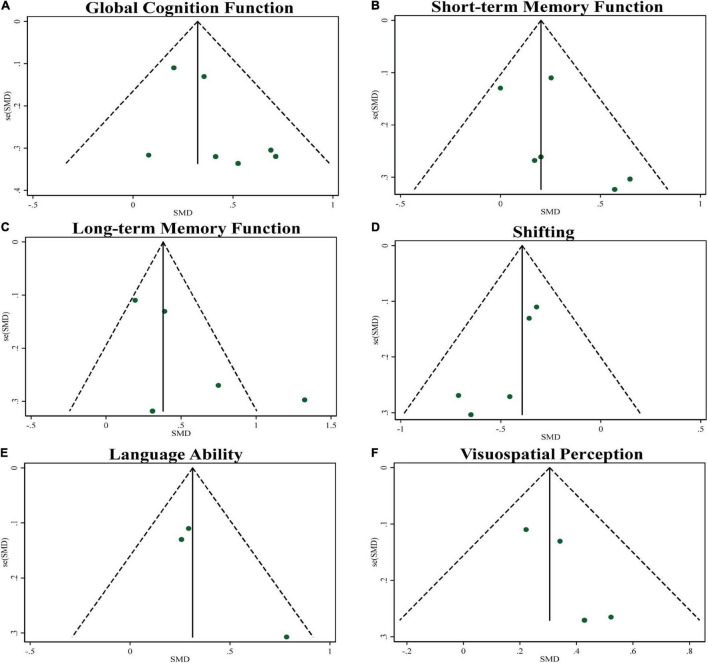
Funnel plots of publication bias for global cognitive function and specific cognitive domains.

The results of our meta-analysis demonstrated that TCE interventions induced a significant improvement in global cognitive function in older adults with MCI. The pooled effect size was significant with small ES (SMD = 0.32, 95% CI 0.18–0.47, *p* < 0.001, Table 3) and without heterogeneity (*I*^2^= 0%, *p* = 0.517). The funnel plot (Figure 2A) and Egger’s test (*t* = 1.72, *p* = 0.147) indicated that there was no publication bias on these results.

**TABLE 3:** Overall and subgroup analysis results regarding the effects of Traditional Chinese Exercises on cognition function.

Outcomes	Overall and Subgroup analysis	No. of studies	SMD (95%CI)	*P*-value	Test of heterogeneity		
					χ^2^	*P*-value	*I*^2^ (%)
Global cognition function	Overall	7	0.32 (0.18, 0.47)	<0.001	5.21	0.517	0
	TCEs vs. Active control	6	0.29 (0.15, 0.44)	<0.001	3.60	0.608	0
	TCEs vs. Inactive control	3	0.58 (0.21, 0.94)	0.002	0.29	0.867	0
	Tai Chi	4	0.36 (0.15, 0.56)	0.001	4.17	0.243	28.1
	Qigong	3	0.33 (−0.04, 0.70)	0.077	1.04	0.595	0
	<24 weeks	4	0.35 (0.06, 0.64)	0.017	4.53	0.210	33.8
	≥24 weeks	3	0.38 (0.16, 0.61)	0.001	0.23	0.890	0
	Educational level < 6 years	3	0.31 (0.12, 0.51)	0.002	2.61	0.271	23.5
	Educational level ≥ 6 years	4	0.43 (0.12, 0.74)	0.007	2.05	0.563	0
	Group practices	5	0.49 (0.21, 0.77)	0.001	2.68	0.613	0
	Group and Home practices	2	0.27 (0.10, 0.43)	0.001	0.79	0.375	0
	Petersen diagnostic criteria	5	0.32 (0.17, 0.47)	<0.001	3.11	0.539	0
	Neuropsychological test diagnostic criteria	2	0.39 (−0.23, 1.02)	0.214	2.00	0.158	49.9
Short-term memory function	Overall	6	0.22 (0.05, 0.39)	0.013	6.13	0.294	18.4
	TCEs vs. Active control	4	0.22 (−0.01, 0.45)	0.056	5.10	0.165	41.1
	TCEs vs. Inactive control	3	0.28 (−0.04, 0.60)	0.082	1.02	0.600	0
	<24 weeks	3	0.29 (0.10, 0.47)	0.003	1.61	0.447	0
	≥24 weeks	3	0.14 (−0.15, 0.43)	0.342	2.79	0.248	28.3
	Educational level < 6 years	2	0.14 (−0.11, 0.38)	0.282	2.23	0.136	55.1
	Educational level ≥ 6 years	4	0.36 (0.08, 0.64)	0.011	2.19	0.534	0
Long-term memory function	Overall	5	0.53 (0.20, 0.86)	0.002	14.90	0.005	73.1
	TCEs vs. Active control	3	0.27 (0.11, 0.43)	0.001	1.40	0.496	0
	TCEs vs. Inactive control	3	0.83 (0.31, 1.34)	0.002	4.73	0.094	57.8
	<24 weeks	2	0.42 (−0.12, 0.95)	0.126	3.63	0.057	72.4
	≥24 weeks	3	0.65 (0.07, 1.23)	0.028	8.74	0.013	77.1
	Educational level < 6 years	2	0.28 (0.09.0.47)	0.004	1.33	0.249	24.7
	Educational level ≥ 6 years	3	0.80 (0.25, 1.36)	0.005	5.52	0.063	63.7
Shifting^§^	Overall	5	−0.39 (−0.54, −0.25)	<0.001	2.66	0.615	0
	TCEs vs. Active control	3	−0.36 (−0.52, −0.20)	<0.001	1.03	0.596	0
	TCEs vs. Inactive control	2	−0.58 (−0.96, −0.21)	0.002	0.45	0.502	0
	<24 weeks	3	−0.45 (−0.70, −0.20)	<0.001	2.53	0.283	20.8
	≥24 weeks	2	−0.38 (−0.61, −0.15)	0.001	0.10	0.747	0
	Educational level < 6 years	2	−0.34 (−0.50, −0.17)	<0.001	0.05	0.828	0
	Educational level ≥ 6 years	3	−0.60 (−0.92, −0.29)	<0.001	0.48	0.785	0
Language ability	Overall	3	0.32 (0.13, 0.51)	0.001	2.56	0.279	21.7
	<24 weeks	2	0.45 (0.00, 0.90)	0.049	2.25	0.133	55.6
	≥24 weeks	1	0.26 (0.00, 0.51)	0.048	0	–	0
Visuospatial perception	Overall	4	0.31 (0.15, 0.46)	<0.001	1.53	0.675	0
	TCEs vs. Active control	2	0.27 (0.11, 0.44)	0.001	0.49	0.482	0
	TCEs vs. Inactive control	2	0.48 (0.11, 0.85)	0.012	0.06	0.804	0
	<24 weeks	2	0.27 (0.05, 0.50)	0.017	1.10	0.295	8.7
	≥24 weeks	2	0.36 (0.15, 0.46)	0.002	0.08	0.773	0

*TCEs, Traditional Chinese Exercises. ^§^Negative effect sizes of shifting indicate greater improvement induced by TCEs.*

**FIGURE 3:**
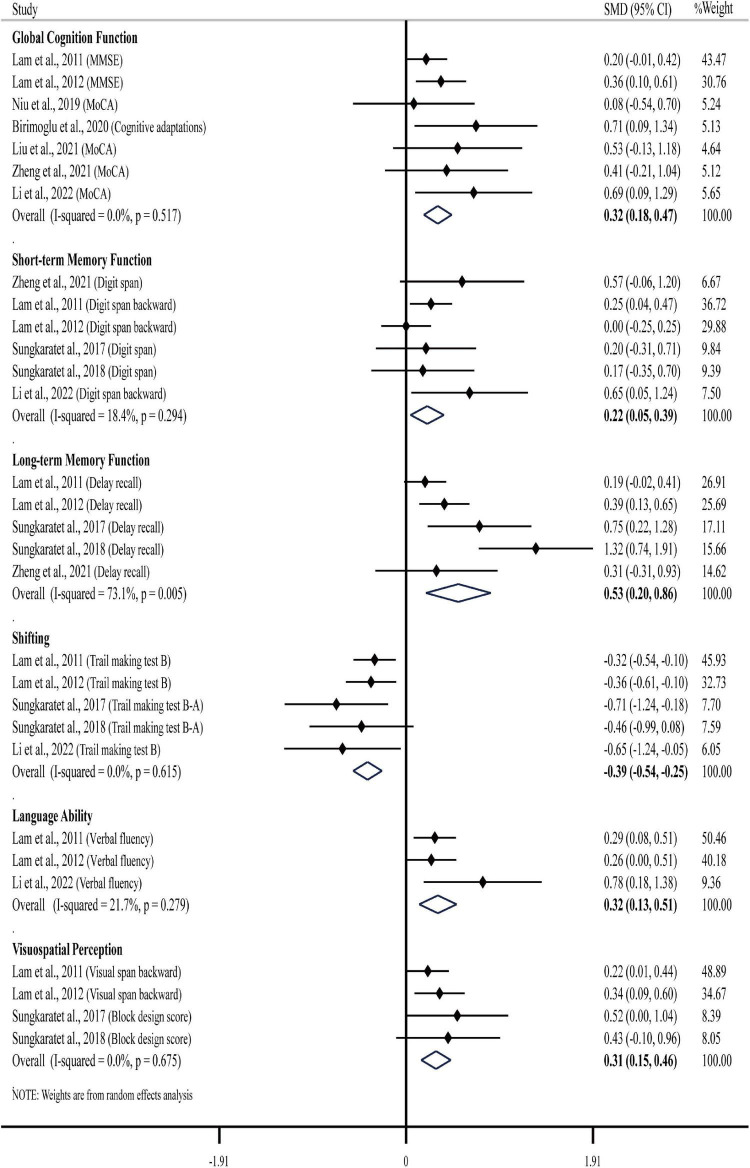
Overall analysis results regarding the effects of TCEs on global cognition function and specific cognitive domains.

The sub-group analysis showed that the effect size of TCEs was significant and small (SMD = 0.29, 95% CI 0.15–0.44, *p* < 0.001) without heterogeneity (*I*^2^ = 0%, *p* = 0.608) when compared to the active control and was moderate (SMD = 0.58, 95% CI 0.21–0.94, *p* = 0.002, Figure 3) without heterogeneity (*I*^2^ = 0%, *p* = 0.867) compared to the inactive control. The sub-group analysis based upon the types of TCEs demonstrated a small effect size for Tai Chi (SMD = 0.36, 95% CI 0.15–0.56, *p* = 0.001) and Qigong (SMD = 0.33, 95% CI −0.04 to 0.70, *p* = 0.077). Regarding to the length of sessions, there was a small effect size for sessions length greater than 24 weeks (SMD = 0.38, 95% CI 0.16–0.61, *p* = 0.001), while the effect size of less than 24 weeks was small (SMD = 0.35, 95% CI 0.06–0.64, *p* = 0.017). Regarding to the education level of participants, there was a close to moderate effect size for education length greater than 6 years (SMD = 0.43, 95% CI 0.12–0.74, *p* = 0.007), while the effect size of less than 6 years (SMD = 0.31, 95% CI 0.12–0.51, *p* = 0.002). Regarding to the intervention environment, the results revealed small effect sizes for both group practices (SMD = 0.49, 95% CI 0.21–0.77, *p* = 0.001) and group practices plus individual home workouts (SMD = 0.27, 95% CI 0.10–0.43, *p* = 0.001). Regarding to the diagnostic criteria for MCI, the results revealed small effect sizes for both Petersen diagnostic criteria (SMD = 0.32, 95% CI 0.17–0.47, *p* < 0.001) and neuropsychological test alone (SMD = 0.39, 95% CI −0.23 to 1.02, *p* = 0.05).

### Effects of Traditional Chinese Exercises on Specific Cognitive Domains

#### Short-Term Memory Function

Five studies showed that TCEs (Tai Chi or Baduanjin) cannot induce significantly improvement in digit span test (Lam et al., 2011, 2012; Sungkarat et al., 2017, 2018; Zheng et al., 2021). One study did not report the statistical significance of the digit span test (Li et al., 2022).

The results of our meta-analysis demonstrated that TCE interventions induced a significant improvement in short-term memory function in older adults with MCI. The pooled effect size was significant with small ES (SMD = 0.22, 95% CI 0.05–0.39, *p* = 0.013, Table 3) and low heterogeneity (*I*^2^ = 18.4%, *p* = 0.294). The funnel plot (Figure 2B) and Egger’s test (*t* = 1.29, *p* = 0.266) indicated that there was no publication bias on these results.

The sub-group analysis showed that the effect size of TCEs was small (SMD = 0.22, 95% CI −0.01 to 0.45, *p* = 0.056) with low heterogeneity (*I*^2^ = 41.1%, *p* = 0.165) when compared to the active control and was small (SMD = 0.28, 95% CI −0.04 to 0.60, *p* = 0.082) without heterogeneity (*I*^2^ = 0%, *p* = 0.600) compared to the inactive control. The sub-group analysis revealed small effect sizes for both longer duration (≥24 weeks, SMD = 0.14, 95% CI −0.15 to 0.43, *p* = 0.342) and short-to-moderate duration (<24 weeks, SMD = 0.29, 95% CI 0.10–0.47, *p* = 0.003). Regarding to the education level of participants, there was a small effect size for education length greater than 6 years (SMD = 0.36, 95% CI 0.08–0.64, *p* = 0.011), while the effect size of less than 6 years was small (SMD = 0.14, 95% CI −0.11 to 0.38, *p* = 0.282).

#### Long-Term Memory Function

There were inconsistent results when comparing the effects between TCEs and control groups (i.e., health education or stretching). Specifically, three studies showed that Tai Chi can induce significantly improvement in delay recall test (Lam et al., 2012; Sungkarat et al., 2017, 2018); two studies showed that TCEs (Tai Chi or Baduanjin) cannot induce significantly improvement in delay recall test (Lam et al., 2011; Zheng et al., 2021).

The results of our meta-analysis demonstrated that TCE interventions induced a significant improvement in long-term memory function in older adults with MCI. The pooled effect size was significant with moderate ES (SMD = 0.53, 95% CI 0.20–0.86, *p* = 0.002, Table 3) and moderate heterogeneity (*I*^2^ = 73.1%, *p* = 0.005). The funnel plot (Figure 2C) and Egger’s test (*t* = 1.90, *p* = 0.153) indicated that there was no publication bias on these results.

The sub-group analysis showed that the effect size of TCEs was significant and small (SMD = 0.27, 95% CI 0.11–0.43, *p* = 0.001) without heterogeneity (*I*^2^ = 0%, *p* = 0.496) when compared to the active control and was large (SMD = 0.83, 95% CI 0.31–1.34, *p* = 0.002) with moderate heterogeneity (*I*^2^ = 57.8%, *p* = 0.094) compared to the inactive control. Regarding to the length of sessions, there was a moderate effect size for sessions length greater than 24 weeks (SMD = 0.65, 95% CI 0.07–1.23, *p* = 0.028), while the effect size of less than 24 weeks was small (SMD = 0.42, 95% CI −0.12 to 0.95, *p* = 0.126). Regarding to the education level of participants, there was a large effect size for education length greater than 6 years (SMD = 0.80, 95% CI 0.25–1.36, *p* = 0.005), while the effect size of less than 6 years was small (SMD = 0.28, 95% CI 0.09–0.47, *p* = 0.004).

#### Shifting

Two studies showed that Tai Chi can induce significantly improvement in shifting as compared to the health education (Sungkarat et al., 2017, 2018), but inconsistent results were observed when comparing the effects between Tai Chi and stretching (Lam et al., 2011, 2012).

The results of our meta-analysis demonstrated that TCE interventions induced a significant improvement in shifting in older adults with MCI. The pooled effect size was significant with small ES (SMD = −0.39, 95% CI −0.54 to −0.25, *p* < 0.001, Table 3) and without heterogeneity (*I*^2^ = 0, *p* = 0.615). The funnel plot (Figure 2D) and Egger’s test (*t* = −3.78, *p* = 0.033) indicated evidence of asymmetry, but the Trim and Fill method for sensitive analysis showed that the pooled ES (fixed SMD = −0.39 vs. random SMD = −0.39) was robust after filled meta-analysis.

The sub-group analysis showed that the effect size of TCEs was significant and small (SMD = −0.36, 95% CI −0.52 to −0.20, *p* < 0.001) without heterogeneity (*I*^2^ = 0, *p* = 0.596) when compared to the active control and was moderate (SMD = −0.58, 95% CI −0.96 to −0.21, *p* = 0.002) without heterogeneity (*I*^2^= 0%, *p* = 0.502) compared to the inactive control. Regarding to the length of sessions, the results revealed small effect sizes for both longer duration (≥24 weeks, SMD = −0.38, 95% CI −0.61 to −0.15, *p* = 0.001) and short-to-moderate duration (<24 weeks, SMD = −0.45, 95% CI −0.70 to −0.20, *p* < 0.001). Regarding to the education level of participants, there was a moderate effect size for education length greater than 6 years (SMD = −0.60, 95% CI −0.92 to −0.29, *p* < 0.001), while the effect size of less than 6 years was small (SMD = −0.34, 95% CI −0.50 to −0.17, *p* < 0.001).

#### Language Ability

Two studies showed that Tai Chi cannot induce significantly improvement in language ability as compared to the stretching (Lam et al., 2011, 2012).

The results of our meta-analysis demonstrated that TCE interventions induced a significant improvement in language ability in older adults with MCI. The pooled effect size was significant with small ES (SMD = 0.32, 95% CI 0.13–0.51, *p* = 0.001, Table 3) and with low heterogeneity (*I*^2^ = 21.7%, *p* = 0.279). The funnel plot (Figure 2E) and Egger’s test (*t* = 2.94, *p* = 0.208) indicated that there was no publication bias on these results.

The sub-group analysis revealed small effect sizes for both longer duration (≥24 weeks, SMD = 0.26, 95% CI 0.00–0.51, *p* = 0.048) and short-to-moderate duration (<24 weeks, SMD = 0.45, 95% CI 0.00–0.90, *p* = 0.049).

#### Visuospatial Perception

One study showed that Tai Chi can induce significantly improvement in visuospatial perception as compared to the health education (Sungkarat et al., 2017), but inconsistent results were observed when comparing the effects between Tai Chi and control groups (i.e., health education or stretching) (Lam et al., 2011, 2012, 2014; Sungkarat et al., 2018).

The results of our meta-analysis demonstrated that TCE interventions induced a significant improvement in visuospatial perception in older adults with MCI. The pooled effect size was significant with small ES (SMD = 0.31, 95% CI 0.15–0.46, *p* < 0.001, Table 3) and no heterogeneity (*I*^2^ = 0, *p* = 0.675). The funnel plot (Figure 2F) and Egger’s test (*t* = 2.58, *p* = 0.123) indicated that there was no publication bias on these results.

The sub-group analysis showed that the effect size of TCEs was significant and small (SMD = 0.27, 95% CI 0.11–0.44, *p* = 0.001) without heterogeneity (*I*^2^ = 0%, *p* = 0.482) when compared to the active control and was almost close to moderate (SMD = 0.48, 95% CI 0.11–0.85, *p* = 0.011) without heterogeneity (*I*^2^ = 0%, *p* = 0.804) compared to the inactive control. Regarding to the length of sessions, the results revealed small effect sizes for both longer duration (≥24 weeks, SMD = 0.36, 95% CI 0.15–0.46, *p* = 0.002) and short-to-moderate duration (<24 weeks, SMD = 0.27, 95% CI 0.05–0.50, *p* = 0.017).

#### Adverse Event/Side Effects

No severe adverse events due to TCEs on participants were reported in any of these studies. Only one participant in the control group had a fall with minor injury, which, however, was not related to the intervention (Lam et al., 2011, 2012).

## Discussion

This systematic review and meta-analysis quantitatively assessed the effects of TCEs on the general cognitive function and on the different cognitive domains in older adults with MCI. Nine studies were included and assessed as “good quality.” The results suggested that TCE is one promising strategy to enhance global cognition function, as well as the short-term memory, long-term memory, shifting, language ability, and visuospatial perception in older adults with MCI. The sub-group analysis revealed that TCEs could achieve greater benefit in improving cognitive function as compared to inactive control groups (i.e., routine daily activities), but it was not superior to regular exercise (i.e., low intensity aerobic exercise or stretching). TCEs have great potential to improve the long-term memory function of the older adults with aMCI. The effects of TCEs on long-term memory function may be influenced by the duration of the intervention (i.e., < 24 weeks or ≥ 24 weeks), and the educational level of the participants. The quality in the body of evidence is moderate.

Inconsistent with previous meta-analysis (Zhang et al., 2019) that consisted of only five publications, we here demonstrated that the TCEs can significantly improve the global cognitive function and working memory, shifting, language ability, and visuospatial perception in MCI. This improvement may arise from the enhancement in the underlying elements that contribute the cognitive function, including mood, cardiovascular health, motor fitness, social interaction, and meditation (Wang et al., 2013; Chang et al., 2014; Yeung et al., 2018). Additionally, studies have shown that TCEs are beneficial to the structure and function of the brain regions, including the prefrontal cortex, temporal cortex, hippocampus, and medial prefrontal cortex, all critical to the regulation of cognitive function (Tao et al., 2016; Wu et al., 2018; Yue et al., 2020). TCEs can also significantly enhance the intrinsic functional connectivity between the norepinephrine and dopamine systems (Liu et al., 2021). All of these potential functional improvements together may result in the improvement of cognitive functions in MCI, shedding light into non-pharmacological therapies for older adults with MCI.

It should be noted that the pooled effect sizes of TCEs on cognitive function were small (ES = 0.21∼0.45) when compared to active control groups and/or in the short duration. The results were inconsistent with a previous study which showed Tai Chi appears to have a stronger effect on cognitive gains (Northey et al., 2018). The results may be affected by the duration of intervention, especially long-term memory function. For example, the one study showed that Tai Chi intervention of fewer than 24 weeks could not significantly improve the long-term memory compared to stretching (Lam et al., 2011), but session length greater than 24 weeks could lead to greater benefits in long-term memory (Lam et al., 2012). Previous meta-analysis which suggested Tai Chi intervention of at least 12 weeks could induce large improvements on global cognitive function and long-term memory, but it included eight quasi-experimental studies, and did not consider the selection of the control group as a potential moderator (Wei et al., 2020). The results support the practice guideline recommendations that suggest a possible benefit of 24 weeks exercise for cognition in MCI (Petersen et al., 2018). In addition, the heterogeneity in the education level may potentially contribute to insufficient effects of TCEs (Lam et al., 2011, 2012). The results showed that TCEs may be more beneficial to older adults with higher educational levels to improve their cognitive function. Therefore, future implementation of the TCEs on MCI should take the education background and the length of follow-up period into consideration to explore the optimal design of the TCEs intervention program.

The results showed that effect sizes of Qigong and Tai Chi on global cognition function were both small. This is inconsistent with previous meta-analysis showing that Qigong may be the appropriate TCE for people with cognitive impairment (Li et al., 2021). However, only three studies examined the effects of Qigong on the cognitive function, and no direct comparison between Qigong and Tai Chi was performed. More studies with larger sample size to directly compare the effects between Qigong and Tai Chi are thus highly demanded. Uniquely, two studies (Niu et al., 2019; Li et al., 2022) explored the effect of TCEs (Qigong or Tai Chi) in the combination with cognitive training and observed that this kind of combined intervention induced greater effects than using TCEs or cognitive training only, suggesting it may be helpful to design and implement the combined intervention for the restoration of cognitive function.

The results revealed that the improvement in long-term memory was more prominent. One potential reason is that the vast majority (approximately 87%) of the participants were diagnosed with aMCI which is a syndrome characterized by memory dysfunction. The results focus more on aMCI rather than non-amnestic MCI which impairment of other cognitive features (e.g., language, visuospatial) is more prominent. Unfortunately, due to the heterogeneity of the diagnostic criteria for MCI, we were unable to identify the type of MCI in the participants in the two included studies (Niu et al., 2019; Birimoglu Okuyan and Deveci, 2020). Group practices may be the most effective mode of intervention for TCEs due to its acceptability, safety and sociability. For example, one study found that remotely delivered Tai Chi group practice sessions via videoconferencing to community-based older adults with MCI was very popular (Li et al., 2022). All included studies used the neuropsychological tests to assess the cognitive functions, but no longitudinal data on how the TCEs help reduce the hazards of dementia or other risk events in older adults with MCI are available, which is thus warranted to be examined in future RCTs with long-term follow ups.

### Strengths and Limitations

This systematic review and meta-analysis explicitly examines the effects of TCEs including both Qigong (i.e., Breath Qigong, Baduanjin) and Tai Chi on the cognitive function in older adults with MCI based upon only RCTs with good quality. The sub-group analyses provide a comprehensive scope of the effects of TCEs on cognitive functions and the influence of the design of TCEs protocol on its effects. However, several limitations need to be noted. The results of subgroup analysis should be taken with cautiousness due to very limited number of included studies, suggesting more studies are needed in this field in the near future. Additionally, studies with positive results are more likely to be published, so there may be some publication bias. The difference in the education level of older adults with MCI may lead to heterogeneity of results, which needs to be carefully assessed in the future. Although the sensitivity analysis showed robust results in this meta-analysis, there was still moderate heterogeneity in memory function after subgroup analysis, which may indicate the results here need more caution. Potential participants with non-amnestic MCI may also contribute to bias in the results, suggesting that future studies need to implement a standardized and validated criterion for the screening of MCI. Five out of included studies were completed in China, indicating TCEs were still mainly focused in its origin; Trials consisting of participants across multiple culture and conducted by more groups are thus highly demand, of which the findings will ultimately help the validation of the study findings and the optimal design of TCEs for different cultural cohorts.

## Conclusion

In summary, this comprehensive systematic review and meta-analysis suggests that TCEs has promise to enhance both global cognitive function and multiple domains of cognitive function (i.e., memory, shifting, language ability, visuospatial perception), which needs to be confirmed by studies with more rigorous design. The results of this work provide critical knowledge for the design of future studies implementing TCEs as well as its clinical practice, including the type of TCEs, and the duration length of the intervention. Future RCTs with rigorous designs are needed to help obtain more definitive conclusions on the effects of TCEs on cognitive function in older adults with MCI.

## Data Availability Statement

The original contributions presented in the study are included in the article/Supplementary Material, further inquiries can be directed to the corresponding author/s.

## Author Contributions

KZ and ML performed literature searches, conducted study selection, data extraction, and study quality assessment. JZ and DB provided methodological insight and guidance throughout the process. KZ drafted the manuscript. KZ, JZ, and DB critically revised the manuscript and contributed to the final version of the manuscript. All authors were involved in conceptualizing the study.

## Conflict of Interest

The authors declare that the research was conducted in the absence of any commercial or financial relationships that could be construed as a potential conflict of interest.

## Publisher’s Note

All claims expressed in this article are solely those of the authors and do not necessarily represent those of their affiliated organizations, or those of the publisher, the editors and the reviewers. Any product that may be evaluated in this article, or claim that may be made by its manufacturer, is not guaranteed or endorsed by the publisher.
