# Supplementary material for: Effects of Traditional Chinese Exercises on Cognitive Function in Older Adults With Mild Cognitive Impairment: A Systematic Review and Meta-Analysis
Source: Front Hum Neurosci. 2022 Mar 25;16:849530. doi: 10.3389/fnhum.2022.849530 (PMC8989961; doi:10.3389/fnhum.2022.849530)
Supplement: Supplementary file 1 [file Data_Sheet_1.docx]

Supplementary Materials

# Supplementary Methods 1: Detailed Search Strategy

**Search Strategy**

| Process | Keywords |
| --- | --- |
| # 1 | "Tai chi" OR "Taijiquan" OR "Tai chi quan" OR "Tai chi chuan"OR "Taiji" OR "Baduanjin" OR "Wuqinxi" OR "Yijinjing" OR "Liuzijue" OR "Qigong" OR "Traditional Chinese Exercis*" OR "Traditional Chinese Medicine Exercis*" |
| # 2 | "Cognitive Function" OR "Cognitive Decline" OR "Cognitive Impairment" OR "Mild Cognitive Impairment" OR "Memory Function" OR "Executive Function" OR "Early-stage Dementia" OR "Neurocognitive Disorder*" OR "Mental Deterioration" OR "Neurodegenerative" OR "Alzheimer's Disease" OR "Psychiatric Disorders" OR "Parkinson’s Disease" OR "Cardiovascular Diseases" |
| # 3 | "Older Adults" OR "Elderly" OR "Elder Adults" |
| # 4 | # 1 AND # 2 AND # 3 |

# Supplementary Table 1: The quality of the evidence (GRADE)

| **No of studies** | **Design** | **Risk of bias** | **Inconsistency** | **Indirectness** | **Imprecision** | **Other considerations** | **Absolute Effect (95% CI)** | **Quality** | **Importance** |
| --- | --- | --- | --- | --- | --- | --- | --- | --- | --- |
| **Global Cognition Function** | | | | | | | | | |
| 7 | randomised trials | serious^1^ | no serious inconsistency | no serious indirectness | no serious imprecision | none | SMD 0.32 higher (0.18 to 0.47 higher) | ⊕⊕⊕O  MODERATE | CRITICAL |
| **Short-term Memory Function** | | | | | | | | | |
| 6 | randomised trials | serious^1^ | no serious inconsistency | no serious indirectness | no serious imprecision | none | SMD 0.22 higher (0.05 to 0.39 higher) | ⊕⊕⊕O  MODERATE | IMPORTANT |
| **Long-term Memory Function** | | | | | | | | | |
| 5 | randomised trials | serious^1^ | serious^2^ | no serious indirectness | no serious imprecision | none | SMD 0.53 higher (0.20 to 0.86 higher) | ⊕⊕OO  LOW | IMPORTANT |
| **Shifting** | | | | | | | | | |
| 5 | randomised trials | serious^1^ | no serious inconsistency | no serious indirectness | no serious imprecision | reporting bias^3^ | SMD -0.39 lower (-0.54 to -0.25 lower) | ⊕⊕OO  LOW | IMPORTANT |
|  |  |  |  |  |  |  |  |  |  |
| **Language Ability** | | | | | | | | | |
| 3 | randomised trials | serious^1^ | no serious inconsistency | no serious indirectness | no serious imprecision | none | SMD 0.32 higher (0.13 to 0.51 higher) | ⊕⊕⊕O  MODERATE | IMPORTANT |
| **Visuospatial Perception** | | | | | | | | | |
| 4 | randomised trials | serious^1^ | no serious inconsistency | no serious indirectness | no serious imprecision | none | SMD 0.31higher (0.15 to 0.46 higher) | ⊕⊕⊕O  MODERATE | IMPORTANT |

^1^ Two studies did not report allocation concealment and one study did not report blinding (assessors blinded).
^2^ moderate heterogeneity (*I*^2^ = 73.1%).
^3^ The funnel plot indicated that there may be publication bias on these results.
